# Squamous cell carcinoma of the stomach: focus on a heterogeneous disease at diagnosis. Case report and literature review

**DOI:** 10.3389/fonc.2024.1419923

**Published:** 2024-11-20

**Authors:** Manlio Monti, Francesco Limarzi, Devil Oboldi, Monica Sbrancia, Maria Caterina Pallotti, Giulia Miserocchi, Virginia Ghini, Sofia Zanuccoli, Sara Cagnazzo, Giovanni Luca Frassineti

**Affiliations:** ^1^ Department of Medical Oncology, IRCCS Istituto Romagnolo per lo Studio dei Tumori (IRST) “Dino Amadori”, Meldola, Italy; ^2^ Pathology Unit, “Morgagni-Pierantoni” Hospital, Forlì, Italy; ^3^ Radiology Unit, IRCCS Istituto Romagnolo per lo Studio dei Tumori (IRST) “Dino Amadori”, Meldola, Italy; ^4^ Gastroenterology and Digestive Endoscopy Unit, “Morgagni-Pierantoni” Hospital, Forlì, Italy; ^5^ Gastroenterology and Digestive Endoscopy Unit, “Maurizio Bufalini” Hospital, Cesena, Italy; ^6^ Palliative Care Unit, IRCCS Istituto Romagnolo per lo Studio dei Tumori (IRST) “Dino Amadori”, Meldola, Italy

**Keywords:** primary squamous cell carcinoma of the stomach, gastric cancer, clinical characteristics, criteria for the diagnosis, immunohistochemistry

## Abstract

Primary squamous cell carcinoma (SCC) can originate in different parts of the body, including the head, neck, lung, bronchus, cervix uteri, esophagus, and cardia, and subsequently metastasize to the stomach. Primary gastric squamous cell carcinoma (GSCC) is a rare disease. To better understand GSCC, we present the case of a 72-year-old woman with a primary GSCC. A chest and abdominal CT scan highlighted a 36×26 mm mass with a 41 mm longitudinal diameter, which included the origin of the celiac tripod. The disease appeared to originate exophytically from the gastric wall. An ultrasound-endoscopy showed a hypoechoic formation with not well-defined margins measuring 40×30 mm involving the origin of the celiac tripod, about 10 mm from the gastric wall. An endoscopic fine-needle aspiration showed a poorly differentiated carcinoma. A PET/CT scan showed a hyperaccumulation of the known expansive formation at the celiac tripod (SUV 11.9) without specific cleavage planes from the stomach. A gastroscopy showed a regular esophagus and an absence of gastric protruding lesions. In the subcardial area, on the posterior wall, there was a slightly raised sub-centimetric area covered by bleeding mucosa where the biopsy had been performed. The pathological report showed chronic gastritis. An eco-endoscopy confirmed a hypoechoic neoformation measuring 30×40 mm that appeared to originate from the muscular layer of the gastric wall. The biopsy report was positive for broad-spectrum cytokeratins (AE1/AE3), CK5/6/7, p40, p63 and negative for CK20, PAS, TTF-1, anti-smooth muscle actin, CD45 (LCA), ERG, and S100. The clinical picture suggested poorly differentiated carcinoma with squamous differentiation. We analyzed the main classifications of GSCC cases and compared their characteristics. It is clear that to have an appropriate definition of GSCC, well-defined diagnostic criteria are needed. Currently, there is no consensus. For practical purposes, it would be better to include a panel of CK and p40 to distinguish GSCC from adenocarcinoma. A GSCC outside the mucosa is not rare and could be a true entity.

## Introduction

Primary gastric squamous cell carcinoma (GSCC) is a rare type of gastric cancer. Through the National Cancer Database, Akce et al. collected retrospective data on 61,215 GC cases diagnosed between 2004 and 2013 (1). In their cohort, GSCC represented 1.4% of cases (n=836). The mean age of the patients at diagnosis was 65.9 years (age range, 23 to 90 years). A high percentage of the study population was male (72.5%). On the Surveillance Epidemiology and End Results (SEER) database from 1988 to 2012, GSCC accounted for 0.2% of all primary gastric cancer cases (n=66,372), and 50% of patients with GSCC were diagnosed with stage IV disease (2). The pathogenesis of GSCC is unclear and controversial. GSCC was first identified in 1895 (3), and a decade later, it was described in a second case study (4). To date, about 100 case reports or case series have been reported in the literature. Given the rarity of GSCC, the therapy is also not well-defined and so the prognosis is poor. To date, neither the European Society of Medical Oncology nor the National Comprehensive Cancer Network societies have published recommendations for GSCC. Our case study aims to provide an analysis of the current literature on this diagnosis.

## Case report

In November 2022 a 72-year-old woman went to the emergency room (ER) for atypical chest and epigastric pain, lack of appetite, nausea, vomiting, and a weight loss of about 17 kg in the three months before her ER visit. Her blood count showed the hemoglobin level at 13.9 g/dl (12-18), white blood cells 8.33×10^9^/L, platelets 357×10^9^/L (150-450), International Normalized Ratio 1.04 (0.80-1.20), creatinine 0.60 mg/dl (0.50-1.00), Alanine aminotransferase 10 U/L (<33), total bilirubin 0.34 mg/dl (<1.2), C-reactive protein 1.2 mg/L (<5.0), sodium 137 mMoli/L (136-145), potassium 3.8 mMoli/L (3.5-5.1), and troponin T 9 ng/L (99° percentile), which was stable after three and six hours. The patient had no fever. Additionally, blood pressure and oxygenation parameters were normal. A thorough cardiological assessment with an electrocardiogram was negative.

**Figure 1:**
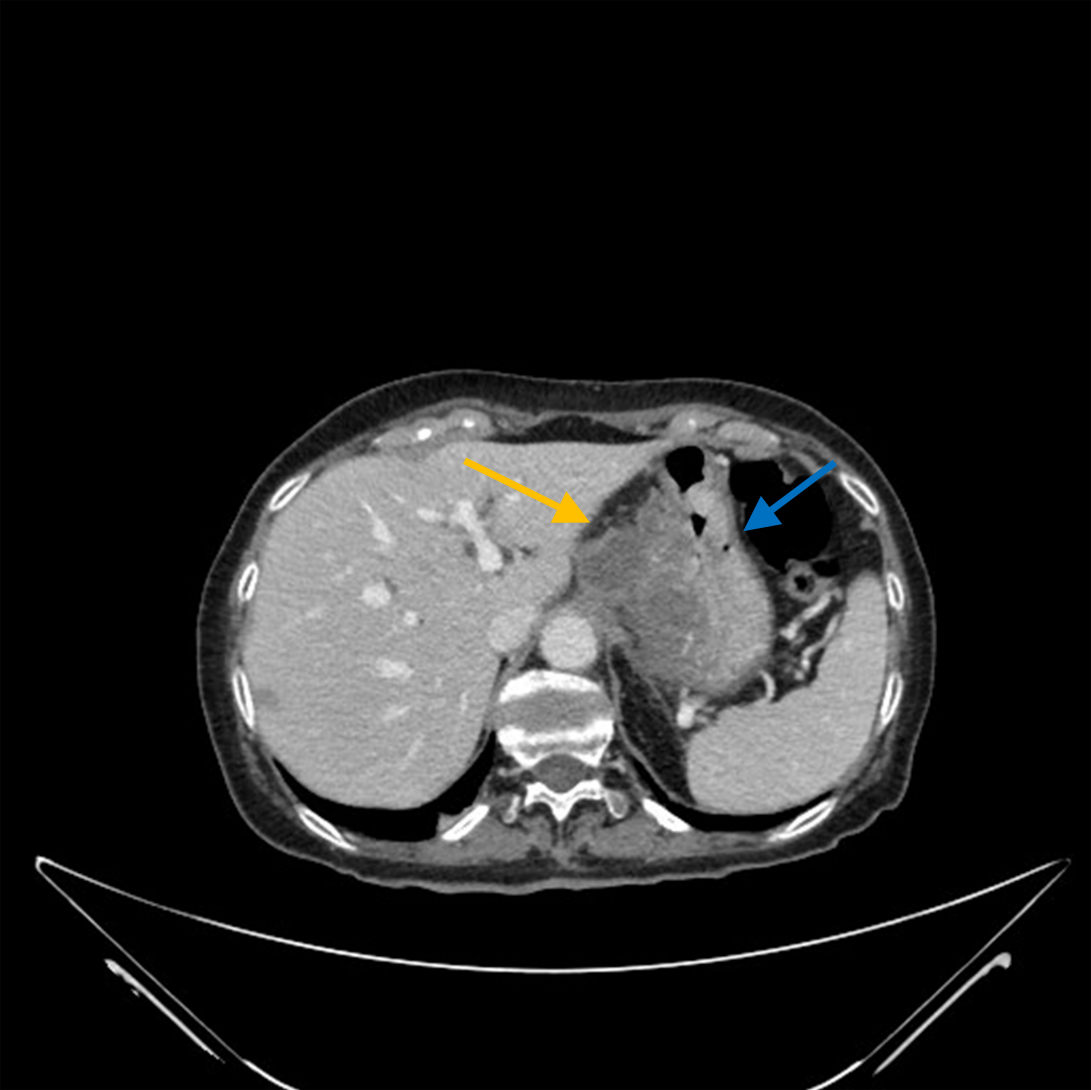
In the abdominal CT scan, the orange arrow shows the neoplastic mass that originates exophytically from the gastric wall. The blu arrow shows the gastric wall.

**Figure 2:**
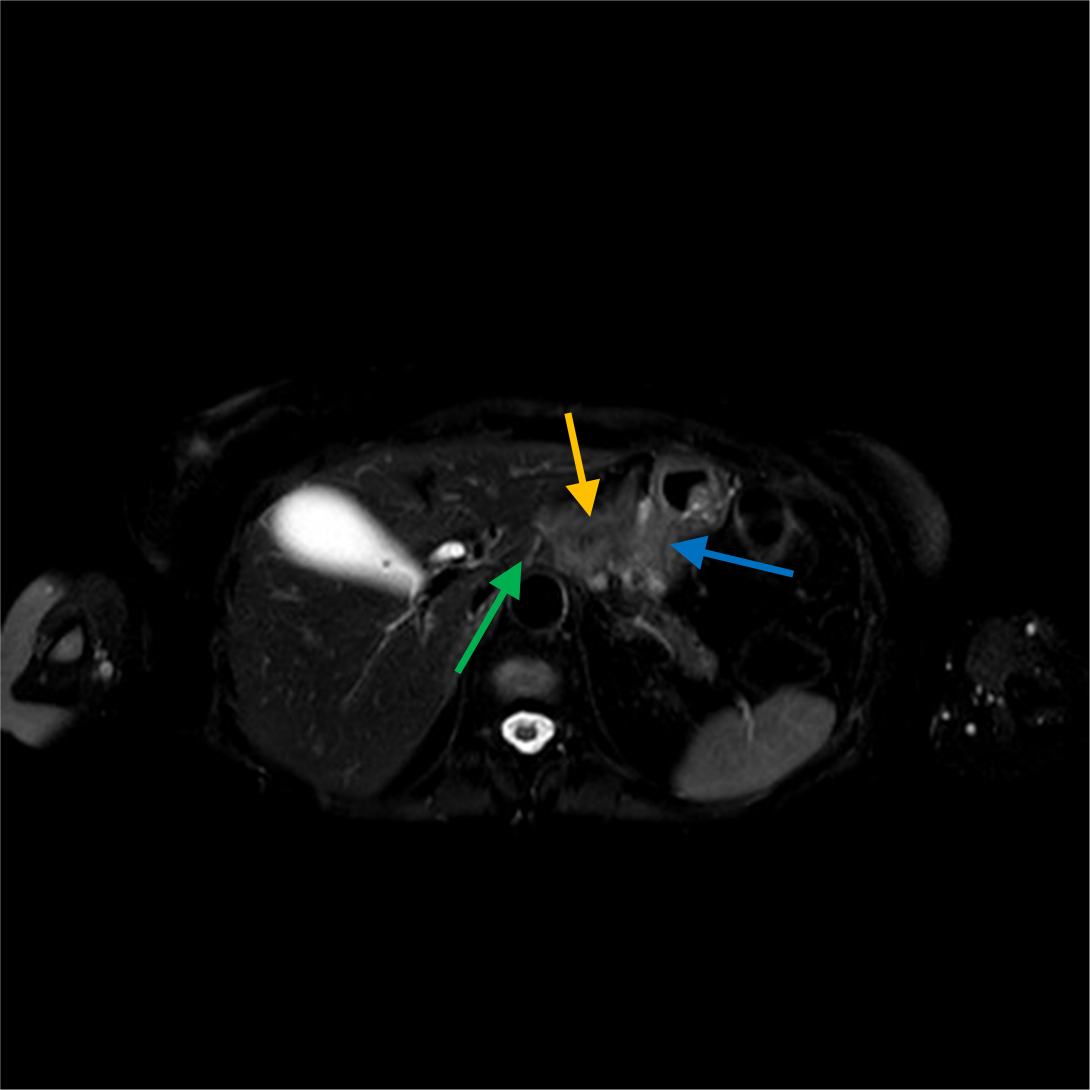
The cNMR confirms the lesion (orange arrow) in correspondence to the celiac tripod (green arrow) with an elongated component to a lesser curvature (blu arrow).

**Figure 3:**
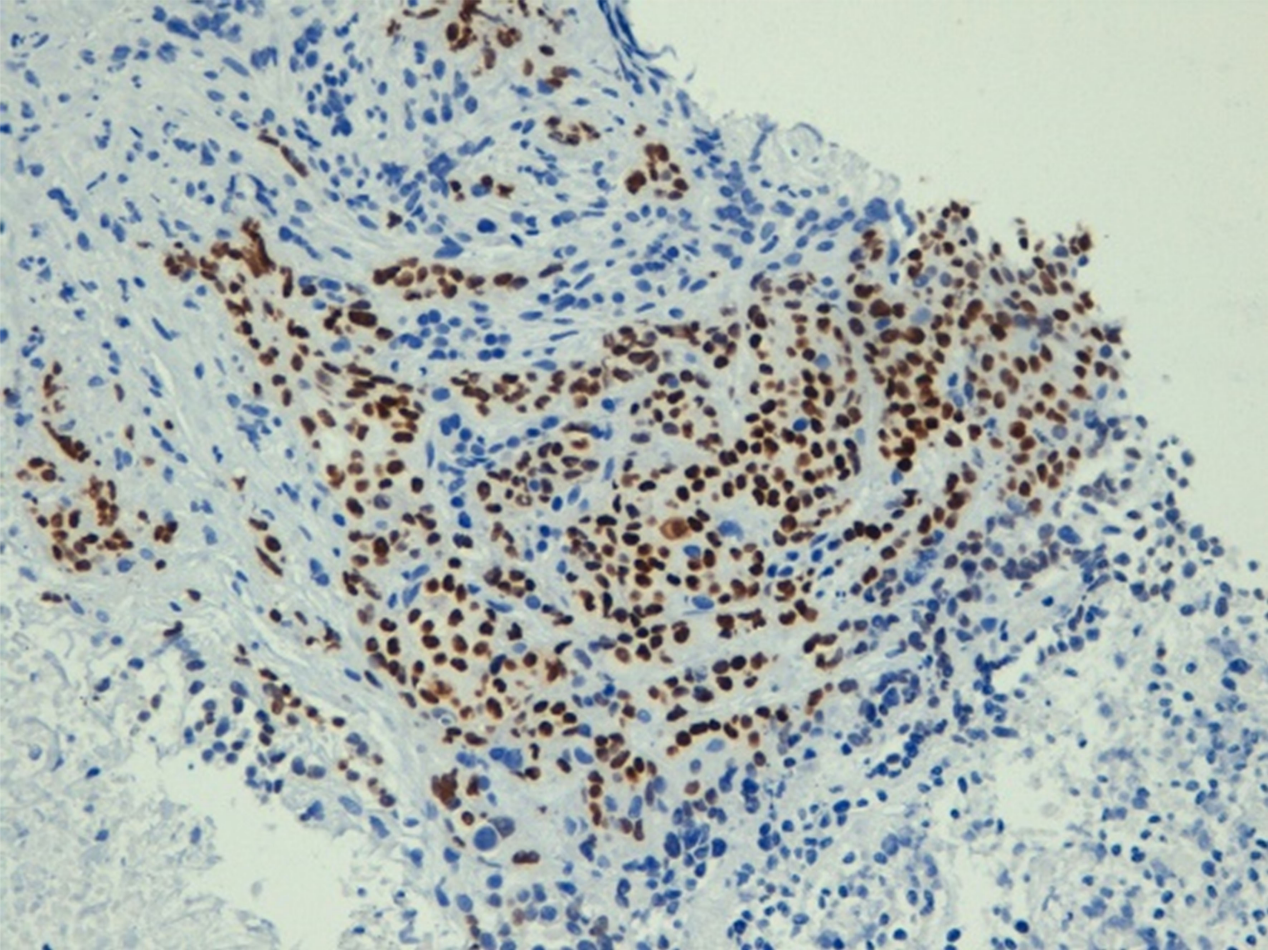
Immunohistochemistry of cancer confirming the expression of p40.

The medical history of the patient reported an appendectomy at 16 years of age and a hysterectomy for uterine fibroid at 60 years of age. The patient was not taking drugs at the time of her ER visit. She was admitted to the Cardiology ward, where a coronarography examination result was negative for stenosis. She was discharged and subsequently had a gastroscopy (**Supplementary Figure S1**), which showed mild gastropathy with signs of atrophy, and biopsies on the corpus and fundus seemed to indicate autoimmune atrophic gastritis. An abdominal ultrasound found a suspected pancreatic hypoechoic oval formation. Because the symptoms persisted, she was admitted to the Medicine ward and administered low osmolarity parental nutrition (amino acids, glucose, lipids, sodium, calcium, potassium, magnesium) of about 1,500 ml with 1,960 total kcal. To treat the pain, she began *oxycodone* hydrochloride 5 mg twice daily and transdermic fentanyl 12 microg/h every three days. The pain-killing drugs were soon increased to a continuous 24-hour intravenous infusion of 80 mg of morphine hydrochloride and 4 mg of dexamethasone administered intravenously twice daily. A chest and abdominal CT scan showed a 36×26 mm mass with a longitudinal diameter of 41 mm, which included the origin of the celiac tripod. The mass seemed to originate exophytically from the gastric wall with an apparent cleavage plane with the head of the pancreas (**Figure 1**). The patient was transferred to the Gastroenterology ward. An ultrasound endoscopy showed a hypoechoic formation with not well-defined margins measuring 40×30 mm incorporating the origin of the celiac tripod, distancing about 10 mm from the gastric wall. The endoscopic fine-needle aspiration reported a poorly differentiated carcinoma positive for CKAE1/AE3, CK7, CK8, CK 19, focally for CK20 and CDX2, whereas it was negative for CD20 and TTF1. The ultrasound-endoscopy described a lymphadenopathy of 12 mm near the bigger lesion. A PET/CT scan showed a hyperaccumulation of the known expansive formation at the celiac tripod (SUV 11.9) without certain cleavage planes from the stomach. There was also hyperaccumulation at the level of the antrum and duodenal gastric passage (SUV 6). A multidisciplinary team requested a second esophagus-gastro-enteroscopy and a nuclear magnetic cholangio resonance (cNMR). The second gastroscopy (**Supplementary Figure S2A**) - performed at another hospital - showed a regular esophagus and no lesions protruding in the stomach. On the posterior wall of the subcardial area, there was a slightly raised subcentimetric area type 0-IIa covered by bleeding mucosa where the biopsy had been performed (**Supplementary Figure S2B**). The remaining mucosa was discolored with areas of atrophy. The immunohistochemistry with CK AE1-AE3 confirmed chronic gastritis of moderate degree in the quiescent phase. The report was negative for Helicobacter Pylori. The cNMR excluded hepatobiliary diseases and confirmed the lesion in correspondence to the celiac tripod with an elongated component to a lesser curvature (**Figure 2**). Given the importance of distinguishing the origin of the primary, a second upper gastrointestinal eco-endoscopy was performed with the pathologist on site while the colonoscopy showed diverticula. The upper eco-endoscopy confirmed a hypoechoic neoformation measuring 30×40 mm, which seemed to originate from the muscular layer of the gastric wall and incorporated the celiac tripod. The lesion appeared to maintain the cleavage plane with the body of the pancreas and the left hepatic lobe. The exam identified many pericentimetric lymphadenopathies in the perilesional area. The results of the pathology report indicated small aggregates of poorly differentiated tumor cells (**Supplementary Figures S3A, B**). The cells were positive for broad-spectrum cytokeratins (AE1/AE3), CK7, and p40 (**Figure 3**). The cells were intensely and diffusely positive for CK5/6 and p63 (**Supplementary Figures S4A, B**) and negative for CK20, PAS, TTF-1, anti-smooth muscle actin, CD45 (LCA), ERG, and S100. The clinical picture suggested poorly differentiated carcinoma with squamous differentiation. Due to the patient feeling increased epigastric pain, an urgent abdominal CT scan was performed the day after the biopsy. The abdominal CT scan showed an approximately 2 cm wall thickness and an extension of approximately 5 cm at the stomach body along the smaller gastric curvature. This time, there was absence of the cleavage plane of the known celiac tripod lesion. In January 2023, she was admitted to the Oncology ward with a 3 ECOG performance status. The patient was referred to us with a weight of 50 kg, persistent epigastric pain, nausea, and vomiting if she tried to eat. The physical exam was negative. At the last biopsy, a HER 2 evaluation was requested, which was 1+, the PD-L1/CPS score was 45, and the mismatch repair (MLH1, MSH2, MSH6, PMS2) was regular. To complete the staging, she did dermatological, gynecological, and Ear-Nose-Throat (ENT) examinations that excluded primitivity cancers. The research for tumor cells in the urine was also negative. The patient had epigastric pain despite 24-hour continuous intravenous infusion with 80 mg of morphine hydrochloride and 4 mg of dexamethasone intravenous twice daily, so the palliativist added 5 mg of midazolam and pregabalin 75 mg one tablet daily. In one day, the patient asked for four and three intravenous rescue doses of morphine hydrochloride 15 mg and paracetamol 1000 mg respectively, too. Because of severe pain and faint Blumberg positive sign, an abdominal CT was requested to exclude any complications due to the previous biopsy. The last CT scan showed a disease of about 73×44 mm growing quickly on the celiac tripod infiltrating liver, the lesser curve of the stomach, and the body of the pancreas. The palliativist increased the intravenous 24-hour continuous infusion to 100 mg of morphine hydrochloride plus 5 mg of midazolam and pregabalin to 100 mg daily. At the end of January 2023, excluding dihydropyrimidine dehydrogenase deficiency, the patient started a chemotherapy treatment with 85 mg/mg^2^ of oxaliplatin on day 1, 200 mg/m^2^ of calcium levofolinate on day 1, 400 mg/m^2^ of 5-fluorouracil bolus on day 1, 2, 400 mg/m^2^ of 5-fluorouracil on day 1 for 48 h (mFOLFOX6). The **Supplementary Figure S5** summarizes the diagnostic and therapeutic steps. About two weeks after the chemotherapy, when it was possible to repeat the cycle of chemotherapy, the health condition of the patient progressively worsened. The patient presented frequent episodes of mental confusion and delirium. She was subsequently transferred to hospice care where she died a few days later.

## Discussion

Given that the gastric mucosa is made of glandular structures, it is not surprising that adenocarcinoma is the most frequent histotype. Other tumors, such as lymphoma, carcinoid, and stromal tumors, are less common. First, Boswell and Helwig defined four histopathologic criteria for the diagnosis of GSCC, of which at least one must be present to make such a diagnosis: 1) keratinized cell masses forming keratin pearls, 2) mosaic cell arrangement, 3) intercellular bridges, and 4) high concentration of sulfhydryl and/or disulfide groups, indicating the presence of keratin or prekerat (5). In 1967, in the light of these alternative pathologies, Parks suggested three criteria for the diagnosis of primary SCC of the stomach: 1) cancer not located in the cardias, 2) cancer not extending into the esophagus, and 3) no evidence of SCC elsewhere in the body (3). In 1999, a more recent diagnostic criteria for GSCC was proposed by the Japanese diagnostic criteria: 1) all the tumor cells are SCC cells and any part that does not contain gland cancer cells, and 2) there is sufficient evidence to show that SCC originates in the gastric mucosa (6).

In 1969, Straus et al. proposed potential pathogenesis sources of GSCC: 1) squamous differentiation of adenocarcinoma with complete replacement of glandular elements, 2) squamous metaplasia of the gastric mucosa before malignant transformation, 3) SCC arising from the vascular endothelium of the stomach, 4) islands of ectopic squamous cells in gastric mucosa, 5) totipotent stem cells in the gastric mucosa capable of differentiating into cells of any type (7).

In 2000, squamous cell extension into the proximal stomach was recognized as a new mucosal abnormality with unknown clinical significance. This mucosal abnormality may represent an esophageal mucosal response to proximal gastric injury (8). In the literature there are reports of primary GSSC on the cardias (9–13), and these are in contrast with Parks’ criteria. These cases could be connected to squamous mucosa in the stomach. It is possible to find squamous mucosa in the stomach, in particular, a complete replacement of normal gastric mucosa by squamous epithelium has been reported in patients with syphilis (14) and after severe stomach injury due to ingestion of a corrosive agent (15). The Boswell and Helwig hypothesis suggested that squamous metaplasia was responsible for malignant transformation (5). Choi et al. described the origin of GSSC as starting with chronic inflammation associated with Mènètrier disease and moving on to squamous metaplasia (16). Mori et al. reexamined three primary stomach tumors, which had been diagnosed as pure GSSC. In each tumor, histological studies revealed minute areas of adenocarcinoma, in addition to large areas of squamous cell carcinoma. This finding led them to suggest that the precursor metaplastic squamous cell lesions may develop from an adenocarcinoma (17). In any case, what Mori et al. described did not agree with the first point of the Japanese diagnostic criteria. Some authors suggested that primary GSCC is related to Human Papilloma Virus, Epstein Barr Virus, and Helicobacter pylori (HP) infections. The HP was excluded in our case. Under chronic inflammatory exposure, tissue stem cell transformation causes epithelial metaplasia and dysplasia, then epithelial carcinoma occurs (18).

In our opinion, it is interesting to focus on the diagnostic heterogeneity of GSSC disease. To do this, we considered: 1) the presence of cancer on the gastric mucosa (as part of Japanese classification), 2) the macroscopic growth site, 3) adhesion to the diagnostic criteria by Parks, 4) the description of metaplasia as well as possible explanation of pathogenesis, 5) the immunohistochemistry (IHC). We believe that comparing the characteristics of GSCC through published case reports can provide insights into its pathogenesis and diagnosis (**Table 1**). Considering the presence of cancer on the stomach mucosa and then the adhesion to the Japanese criteria (6), we found that several cases of GSCC do not respect it (9, 19–33). Probably the Japanese criteria are very restrictive to follow and probably collect the clearest cases of GSSC. Furthermore, there are several reports with exophytic growing (9, 19–25, 27, 34) or with wall thickness but without extension to the gastric mucosa (26, 28–33) like our case. Intestinal metaplasia was present in few cases with wall thickness (18, 33, 35–37), while squamous metaplasia, which is a possible sign of malignant transformation, was described in few cases too (5, 12, 13, 16). Cases without exophytic growing had squamous metaplasia or intestinal metaplasia (9, 19–25, 27, 34). It is possible that an exophytic mass, growing predominantly outside the stomach, could originate from a totipotent stem cell in the deep layer of the gastric wall. To date, the origin of the cancer is speculative.

**Table 1:** Comparison of characteristics of the main case reports published on GSCC.

Author	No. of patients	Tumor location/kind of extension	Gastric mucosa involved by cancer	Adhesion to criteria of Parks	Immunoistochemistry	Squamous metaplasia	Intestinal metaplasia	Gastric resection
Boswell	12	–	Yes (2 pts)	Yes	PAS- (12 pts)	Yes (1 pt)	–	Yes 5 (pts)
Danish Kumar	1	Cardia-lesser curvature/exophytic	No	No	CK5/6 +, p63 +	–	–	–
Guangyao Wang	1	Posterior wall/exophytic	No	Yes	CK 5/6 +, p63 +, CKpan and glutathione S transferase π +, (CD)56-, CDX-2-, chromogranin A-, CK20-, CK7-, S100-, Syn-, Villin-, P-glycoprotein-, EGFR-, p53-	–	–	Yes
Hideyuki Wakabayashi	1	Lesser curvature/exophytic	No	Yes	–	–	–	Yes
Jagoda Jakubik	1	Fundus/endophytic	Yes	Yes	p63 +, CK7 -, CK 19/20 -, CDX2 -, PAS -	–	–	Yes
Juan Antonio González-Sánchez	1	Esophagus-fundus-lesser curvature/endophytic	Yes	No	CK5+, CK7+, CK20+, TTF1+, p63+, chromogranin+, synaptophysin+	No	–	Yes
Kehua Zhou	1	Fundus-posterior wall/exophytic	No	Yes	p40+, CK5/6+, CK7+, TTF-1-, CK20-, CDX2-	–	No	No
Xiao Wang	1	Corpus/wall thickness	Yes	Yes	CK5/6+, p63+, CKL-	–	Yes	Yes
Rodrigo De Assis Moraes	1	Antrum-body-corpus	Yes	Yes	AE1/AE3+, CK5/6+, CK7+, p63+	–	–	Yes
Shengqiang Gao	1	Antrum-corpus/ wall thickness	No	Yes	–	–	–	Yes
Sun Hwi Hwang	1	Fundus/exophytic	No	Yes	–	No	–	Yes
Xu-Dong Wu	1	Posterior wall/exophytic	No	Yes	–	–	–	Yes
Ying Meng	7	4 lesser curvature, 2 greater curvature, 1 diffuse	Not well specified	Yes	–	–	–	No
Yuki Katsura	1	Corpus-posterior wall/ wall thickness	No	Yes	CK5/6+, p40+	No	–	Yes
Yukinori Yamagata	1	Posterior wall of body/ wall thickness	Yes	Yes	AE1/AE3+, CK5/6+, CDX2+, CK20+/-, p63-, p40-, CK7-, synaptophysin-, α-fetoprotein-	No	Yes	Yes
Wolf von Waagner	1	Fundus/wall thickness	No	Yes	CK5/6+, p63+, CD117-, CK20-, p16-	–	–	Yes
Guzman Rojas	1	Corpus-lesser curvature/ Endophytic	Yes	Yes	CK5/6+, p63+, CK7-, CK20-	–	–	Yes
Segura	1	Fundus/wall thickness	Yes	Yes	p63+	No	Yes	No
Hara	1	Greater curvature/exophytic	No	Yes	–	–	–	Yes
Tokuhara	1	Lesser curvature/ wall thickness	Yes	Yes	–	–	–	Yes
Vailas	1	Fundus/wall thickness	Yes	Not specified	AE1/AE3+, p40+, Chromogranin A−, Synaptophysin−, c-erB-2/HER2−CK14+, p53+, CK 5/6+, p63+, MUC1+, PAS-	–	–	Yes
Gülçiçek	1	Antrum-posterior wall/ wall thickness	Yes	Yes	p63+, CK 5/6+ and CK7+	–	Yes	Yes
Yang Chen	21	7 body, 6 fundus, 7 cardias, 1 antrum	Not well specified	No (6 pts)	p63+, CK 5/6+ (4 pts)	–	–	Yes (15 pts)
Yeon Soo Chang	1	Anastomotic site/ wall thickness	No	Yes	p63+, CK 5/6+, CK7-, CK20-	–	–	Yes
Callacondo-Riva	1	Antrum/wall thickness	No	Yes	p63+, CK 5/6+,	No	Yes	Yes
Choi	1	Greater curvature/ wall thickness	Not well specified	Yes	–	Yes	–	Yes
Marubashi	1	Lesser curvature-posterior wall-antrum/wall thickness	Not well specified	Yes	CK 5/6+, CK8+, CK17+, CK19+	No	–	Yes
Schwab	1	Anterior and posterior walls lesser curvature/ wall thickness	No	Yes	–	No	–	Yes
Karaka	1	Lesser curvature/ wall thickness	Not well specified	Yes	–	–	–	Yes
Won Lee	2	Antrum-body	Yes	Yes	p53+, p16 -	No	–	Yes (2 pts)
Lei Gao	1	Antrum/exophytic	No	Yes	p63+, CK 5/6+, CD117-	–	–	Yes
Little	1	Antrum/exophitic	No	Yes	AE1/AE3+, CK5+, p63+	–	–	Yes
Mardi	1	Antrum/wall thickness	No	Yes	CK5/6+, CK10+, CK19+, CK14+, CK7-, CK20-	–	–	Yes
Modi	1	Lesser curvature/ Endophytic	Yes	Yes	–	–	–	Yes
Oono	1	Cardias	Yes	No	–	Yes	–	Yes (ESD)
Patnayak	1	Antrum/wall thickness	Yes	Yes	–	No	–	Yes
Raju	1	Posterior wall/ wall thickness	Not well specified	Yes	–	–	–	No
Yildirim	1	Antrum/exophytic	Not well specified	Yes	–	No	–	Yes
Dursun	1	Corpus/antrum/lesser curvature/wall thickness	Yes	Yes	–	–	–	No
Takita	1	Cardia/Lesser curvature	Yes	No	p53-	Yes	–	Yes
Our case	1	Posterior wall/ Exophytic	No	Yes	AE1/AE3 +, CK5/6+, CK7 +, CK8+, p40+, p63+, CK19+, CDX2+, CK20 -, PAS-, CD 45 (LCA) -, TTF-1 -, ERG-, S100 -, smooth muscle actin -	No	–	No

Pts, patients; ESD, endoscopic submucosal dissection.

Considering the critical issues mentioned, it is important to note there are cancers of unknown primary origin (CUPs). These heterogeneous tumors manifest in a variety of ways and are classified microscopically: well-moderately differentiated adenocarcinomas, poor differentiated adenocarcinomas/undifferentiated, and SCC (38). The study by Guangyao Wang described an interesting case report of a cancer located in the interspace between the liver and the stomach. It involved the serosal fibrous tissue, lamina muscularis, and submucosa of the gastric wall and it had already metastasized to a regional lymph node at the time of surgery. The authors preferred to define the GSCC as an unknown primary site (19).

IHC represents a routine to localize the primary site of metastatic carcinomas and to classify primary tumors (e.g., SCC vs. adenocarcinomas). By using lineage-specific markers, IHC can also be helpful in the setting of poorly differentiated/undifferentiated malignancies. An initial panel of markers should be done to identify the lineage of tumor: carcinoma (cytokeratin AE1/3, OSCAR, CAM5.2), lymphoma (CD20, CD3), melanoma (S-100 protein, SOX10), and sarcoma (desmin, smooth muscle actin, MDM2, ERG). A typical panel includes CK7 and CK20. Usually, the CK7 and CK20 phenotype in combination with one or more additional site-specific markers is sufficient to localize the primary location. For poorly differentiated carcinomas with no clear gland formation, markers of squamous differentiation (p63, p40, CK5/6), neuroendocrine markers (synaptophysin, chromogranin), urothelial markers (GATA3, p63), and renal markers (PAX8) may be indicated based on the available clinical history and morphology of the tumor (39). Distinguishing poorly differentiated adenocarcinoma from SCC is a frequent dilemma, especially in the esophagus. Di Maio et al. evaluated a panel of markers to distinguish esophageal SCC and adenocarcinoma. They found that CK5/6 (sensitivity 98%, specificity 87%) and p63 (sensitivity 100%, specificity 90%) were able to identify the majority of the SCCs. The authors concluded that the lack of CK 5/6 and p63 immunoreactivity excludes SCC and supports the diagnosis of adenocarcinoma (40). Bishop et al. demonstrated the superiority of p40 over p63 in the diagnosis of SCC, especially in the lung (41). Only a few studies compared the expression of p63 and p40 in carcinoma of the gastrointestinal tract. One study compared the expression of p40, p63, and CK5/6 in a group of SCC (n=25) and adenocarcinomas (n=24) across several gastrointestinal tract primaries. P63 was expressed in all SCCs and 12.5% of adenocarcinomas, whereas p40 was expressed in 92.5% of SCCs but only 4.1% of adenocarcinomas (42). Finally, CK5/6 was positive in 96.2% of SCC and 20.9% of adenocarcinomas. Based on this study, it appears that p63 is a more sensitive marker of squamous differentiation while p40 is more specific.

A limitation of our study is that there is a lack of consistency in the IHC analysis across the studies presented in **Table 1** (5, 9–11, 18, 19, 21, 23, 24, 28, 29, 31–33, 35, 37, 43–48), while in other cases the description of IHC is unclear (12, 16, 20, 22, 25–27, 30, 34, 49–55).

In our study, the immunohistochemical findings of CK +, S100-, and LCA- with the presence of AE1/AE3 + led to an epithelial lineage. The smooth muscle actin and ERG - exclude the soft tissue origin. In our case, PAS - contrasted with adenocarcinoma, and the presence of p40 + was compatible with an SCC. To define the squamous origin of the lesion, p40 is more helpful, although not expressed in all cells but the presence of CK5/6 and p63 confirms the squamous differentiation. We are uncertain about the potential pathogenesis of GSCC, although it could originate from a totipotent cell deep in the stomach that quickly extends to lymph nodes. The common characteristic of published case reports, including ours, is that the disease is often localized while CUPs are metastatic malignancies with a primary tumor site that cannot be identified on standard baseline evaluation (56). In this review, most patients underwent gastric resection because the disease was small or locally advanced. However, in our review less than half of the patients operated on had squamous carcinoma that started from the gastric mucosa. This data leads to consider that several cases of GSSC may not arise from the gastric mucosa.

An interesting aspect of this review is that we considered both IHC aspects and the possible site of disease initiation. We believe that some SCCs can originate outside the gastric mucosa and a new classification could be helpful. The hypothetical classification could consider: 1) SCC originating in the stomach, it is possible to be located in the cardias but not extending into the esophagus, 2) no evidence of SCC elsewhere in the body, 3) SCC originates in the gastric mucosa or the wall of the stomach.

Making a diagnosis of gastric infiltrating tumors is challenging and often delayed due to false negative endoscopic and histological tests. Although gastric linitis plastica is the most strongly suspected disease, we had to consider the possibility of other diseases associated with the thickening of the submucosa or muscularis propria, including Menetrie’s gastritis, lymphoid hyperplasia, amyloidosis, gastrointestinal stromal tumor, and malignant lymphoma (57). In our case, as in the case of Lei Gao (23), the first gastroscopic mucosal biopsy showed only inflammation because of the deep originating lesion. The CT scan for GSCC usually shows a heterogeneously enhanced mass. An endoscopic ultrasonography-guided fine-needle biopsy is helpful for the histopathological diagnosis of deep lesions in the gastric wall.

In conclusion, a precise definition of diagnostic criteria is needed to ensure an adequate definition of GSCC, as currently, there is no consensus. For practical purposes, to distinguish SCC from adenocarcinoma, including a panel of CK and p40 could be helpful. We think that GSCC limited to the outside of the mucosa is not rare and could be a real entity.

## Data Availability

The raw data supporting the conclusions of this article will be made available by the authors, without undue reservation.
