# Supplementary material for: Squamous cell carcinoma of the stomach: focus on a heterogeneous disease at diagnosis. Case report and literature review
Source: Front Oncol. 2024 Nov 20;14:1419923. doi: 10.3389/fonc.2024.1419923 (PMC11614721; doi:10.3389/fonc.2024.1419923)
Supplement: Supplementary file 1 [file DataSheet1.pdf]

SUPPLEMENTARY MATERIAL

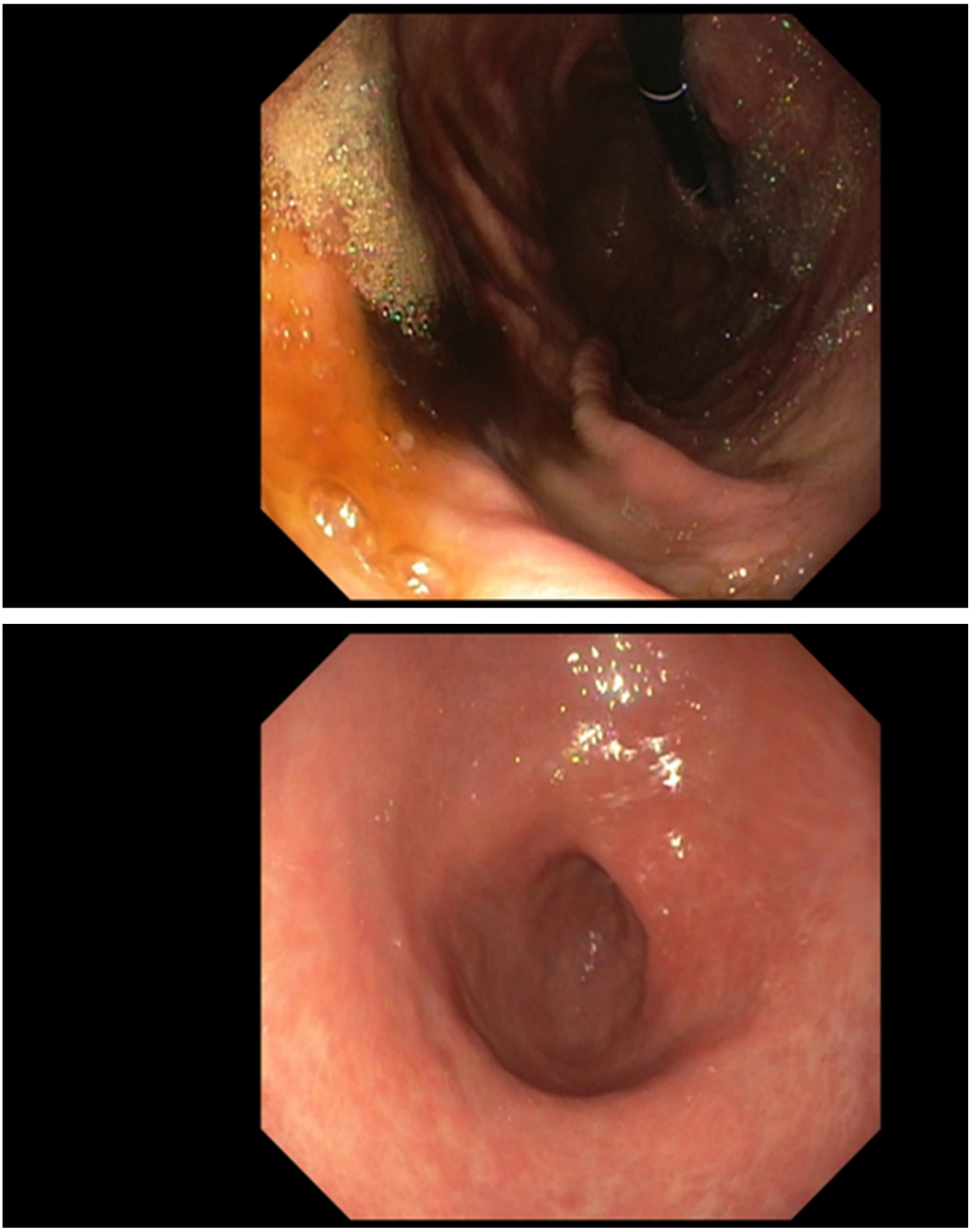

SUPPLEMENTARY FIGURE 1

A first gastroscopy with conventional endoscope was performed. On white-light imaging, no lesions protruding into the stomach were found.

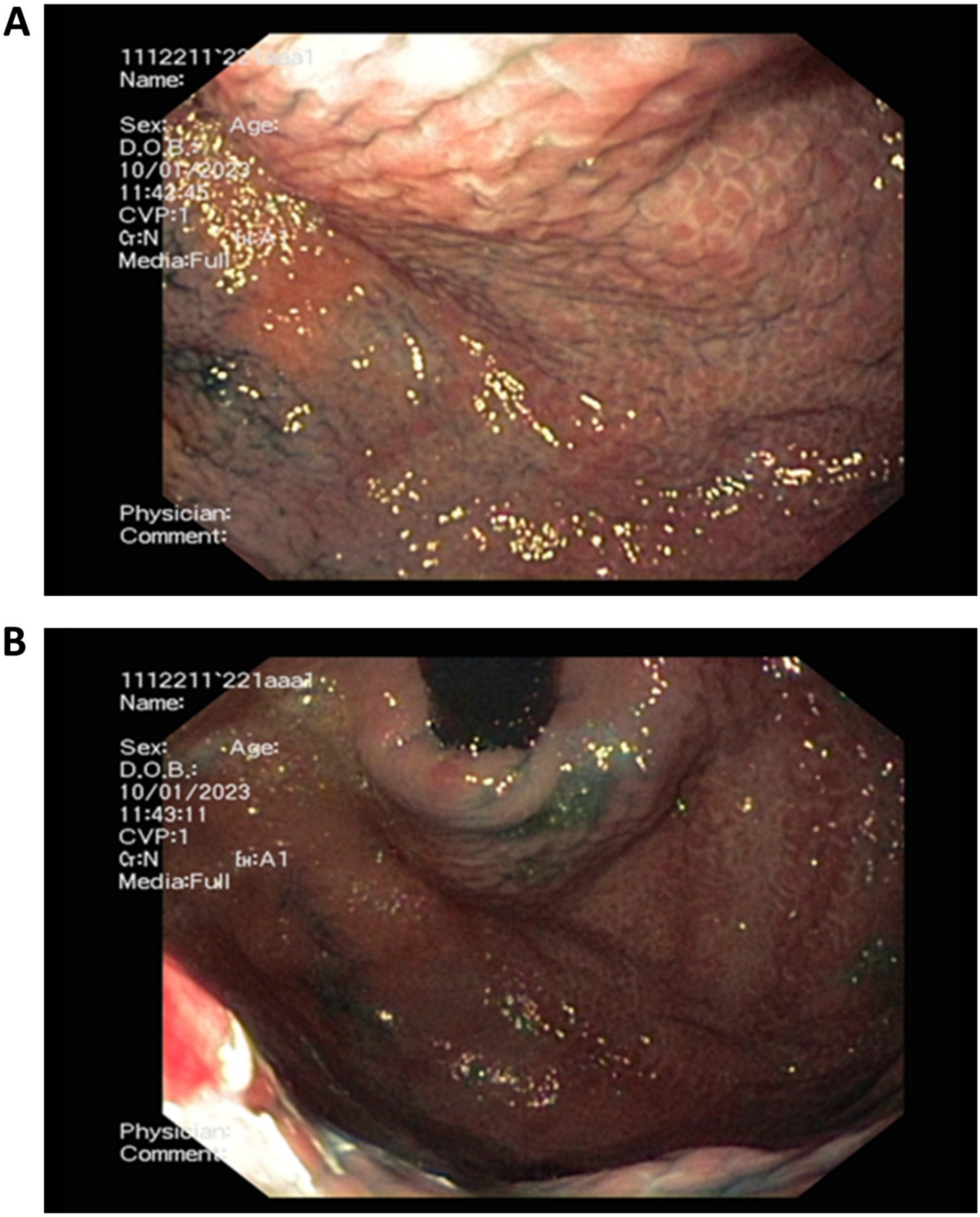

SUPPLEMENTARY FIGURE 2

(A) A second gastroscopy was performed with high-definition (HD) endoscope. After administration of defoaming and mucolytic agents, such as simethicone and acetic acid to enhance mucosal visibility, a traditional chromoendoscopy with indigo carmine was performed. We observe a typical pale appearance of the entire mucosa with increased visibility of submucosal blood vessels due to thinning of the gastric mucosa and loss of gastric fold. **(B)** In the subcardial area, on the posterior wall, we observe a slightly raised sub centimetric area type 0-IIa covered by bleeding mucosa.

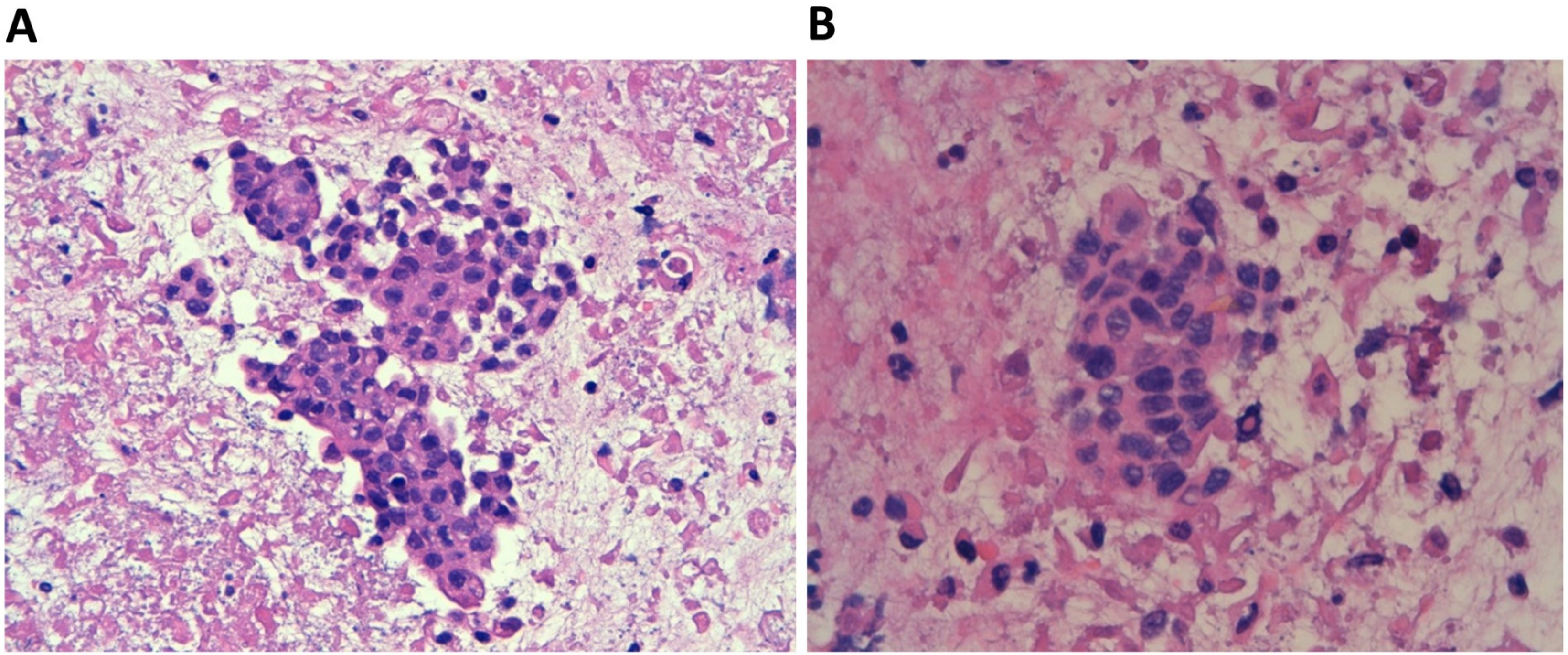

SUPPLEMENTARY FIGURE 3

(A-B) Isolated fragments of cancer cells stained with hematoxylin and eosin (HE) (HE ×400) and (HE ×600).

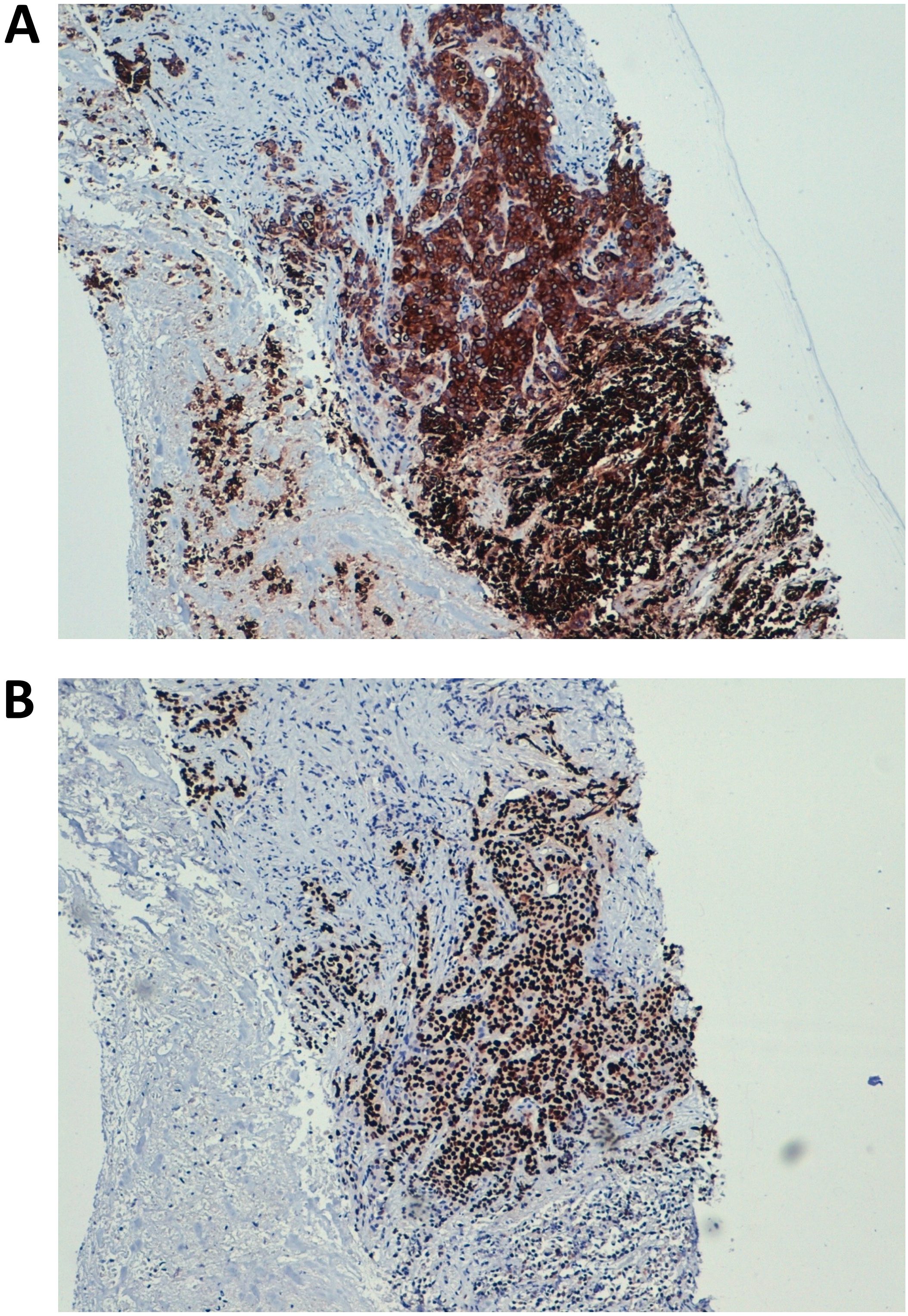

SUPPLEMENTARY FIGURE 4

Immunohistochemical staining was performed for CK5/6 (A) and p63 (B), both of which were intensely and diffusely positive.

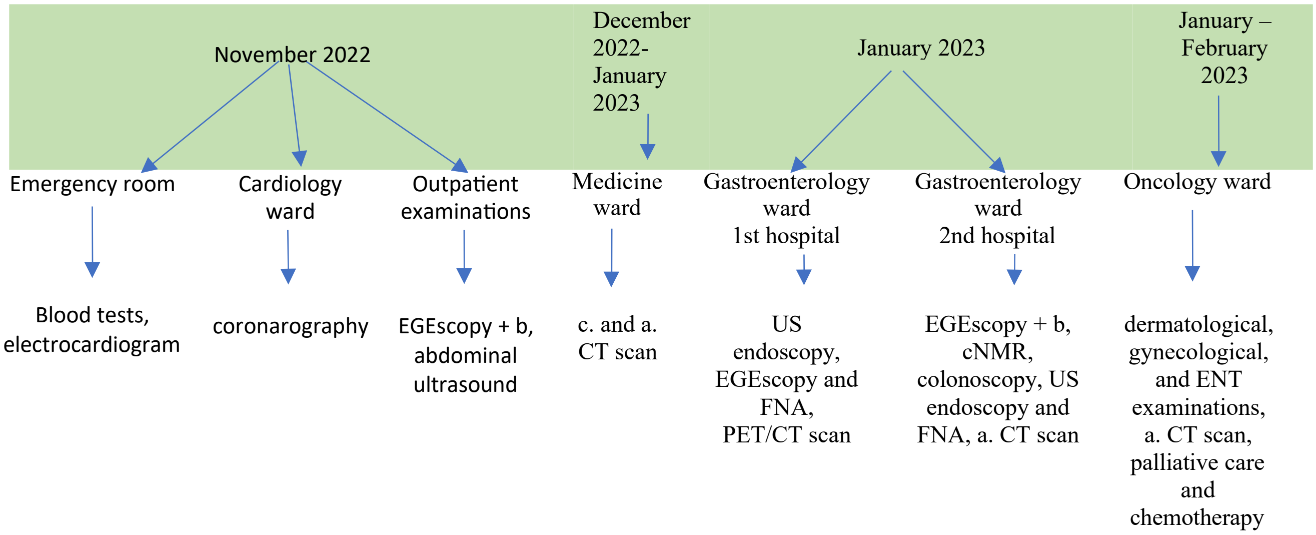

SUPPLEMENTARY FIGURE 5

Timeline of diagnostic and therapeutic steps: esophagus-gastro-enteroscopy (EGEscopy) + biopsy (B), chest (C) and abdominal (A) CT scan, ultrasound endoscopy (US endoscopy), fine-needle aspiration (FNA), magnetic cholangio resonance (cNMR), Ear-Nose-Throat (ENT) examination.
